# Metabolic Characteristics in Meal of Black Rapeseed and Yellow-Seeded Progeny of *Brassica napus*–*Sinapis alba* Hybrids

**DOI:** 10.3390/molecules201219761

**Published:** 2015-11-30

**Authors:** Jinjin Jiang, Yue Wang, Tao Xie, Hao Rong, Aimin Li, Yujie Fang, Youping Wang

**Affiliations:** 1 Jiangsu Provincial Key Laboratory of Crop Genetics and Physiology, Yangzhou University, Yangzhou 225009, China; 18252746287@163.com (Y.W.); 18505143324@163.com (T.X.); 18505143304@163.com (H.R.); yjfang@yzu.edu.cn (Y.F.); 2 Jiangsu Institute of Agricultural Science in the Lixiahe District, Yangzhou 225009, China; yzlam@126.com

**Keywords:** *Brassica napus*, yellow-seeded rapeseed, black-seeded rapeseed, seed meal, metabolites

## Abstract

Breeding of yellow-seeded rapeseed (*Brassica napus*) is preferred over black-seeded rapeseed for the desirable properties of the former. This study evaluated the metabolites and nutritive values of black-seeded rapeseed meal and yellow-seeded meal from the progeny of a *B. napus*–*Sinapis alba* hybrid. Yellow-seed meal presented higher protein (35.46% *vs.* 30.29%), higher sucrose (7.85% *vs.* 7.29%), less dietary fiber (26.19% *vs.* 34.63%) and crude fiber (4.56% *vs.* 8.86%), and less glucosinolates (22.18 *vs.* 28.19 μmol/g) than black-seeded one. Amounts of ash (3.65% *vs.* 4.55%), phytic acid (4.98% *vs.* 5.60%), and total polyphenols (2.67% *vs.* 2.82%) were decreased slightly in yellow-seeded meal compared with black-seeded meal. Yellow-seeded meal contained more essential amino acids than black-seeded meal. Levels of the mineral elements Fe, Mn, and Zn in yellow-seeded meal were higher than black-seeded meal. By contrast, levels of P, Ca, and Mg were lower in yellow-seeded meal. Moreover, yellow-seeded meal showed lower flavonol (kaempferol, quercetin, isorhamnetin, and their derivatives) content than black-seeded meal. Comparison of metabolites between yellow and black rapeseed confirmed the improved nutritional value of meal from yellow-seeded *B. napus*, and this would be helpful to the breeding and improvement of rapeseed for animal feeding.

## 1. Introduction

Rapeseed (*Brassica napus*) is the second most abundant oil crop worldwide next to soybean. Soybean meal is broadly used as an animal fodder for its abundant nutrients, but the supply of soybean meal is insufficient to meet current demands [1]. Rapeseed meal is a potential alternative for animal feed. However, utilization of rapeseed meal as forage has been considerably hindered by its content of antinutrients, which affect its feeding value [2,3]. Consequently, researchers have endeavored to determine the factors influencing the nutritional value of rapeseed meal and proposed methods of meal improvement, such as breeding of yellow-seeded *B. napus* [4,5]. Yellow-seeded *B. napus* is an optimal perspective in breeding of rapeseed, and cultivars/lines with yellow seed coat have been selected from hybridization among *Brassica* species with yellow seed character (*B. juncea*, *B. rapa* and *B. oleracea*) [6]. Introduction of yellow-seed character from relatives in the Brassicaceae (for example, *Sinapis alba*) have been reported [7]. Comparison of metabolites between yellow- and black-seeded *B. napus* has revealed different nutrient contents in the seed meals [8,9,10]. Bell reported the feeding value of rapeseed meal with improved protein and dietary fiber content [11]. Slominski *et al.*, found higher protein (49.8% *vs.* 43.8% dry matter, DM), higher sucrose (10.2% *vs.* 8.8% DM), and less total dietary fiber (24.1% *vs.* 30.1% DM) in meal derived from yellow-seeded *B. napus* than black seed [12]. Antinutrients in seed meal include dietary and crude fibers, as well as polyphenolic compounds, such as hydroxycinnamic acid derivatives and flavonoids. Sinapic acid is the most abundant phenolic compound in rapeseed, and 73% of polyphenols in rapeseed were found to be sinapic acid or its derivatives in conjunction with other chemical groups (e.g., in conjunction with choline to form sinapine). Obied *et al.*, identified 31 polyphenols in canola meal, of which sinapine is the most abundant chemical, comprising 80% of the total phenolics [13]. Sinapine mainly exists in the embryo and contributes to the bitter taste of rapeseed meals. Landero *et al.*, reported that the unpleasant taste of meal with higher sinapine content affected the intake of pigs, which directly reduced their average daily feed intake and average daily weight gain [14,15]. Flavonoids include flavonol and proanthocyanidin (PA), and the relative content of flavonols in rapeseed is lower than that of other compounds, including isorhamnetin, quercetin, kaempferol, and their derivatives. PAs are mainly deposited in the seed coat and tend to be oxidized with proteins and polysaccharides during rapeseed maturation, resulting in a reduction in the nutritional value of seed meal [16]. Jiang *et al.*, reported significant differences in the PA contents of yellow- and black-seeded *B. napus* [7].

Rapeseed with yellow-seeded germplasm does not naturally exist. We obtained a novel yellow-seeded rapeseed from the progeny of *B. napus–S. alba* hybrids, and the oil content of this yellow-seeded rapeseed line (W82) was 6% higher than that of black-seeded rapeseed [7]. In the present study, we compared the metabolites and nutrients between the seed meals of black-seeded rapeseed (*B. napus* cv. Yangyou 6) and yellow-seeded rapeseed (*B. napus–S. alba* hybrid, line W82). This comprehensive comparison will facilitate understanding of the differences in nutritional values between the two rapeseed lines and might contribute to the utilization and molecular dissection of yellow-seeded rapeseed.

## 2. Results and Discussion

### 2.1. Differences in Chemical Composition between Yellow- and Black-Seeded Rapeseed Meals

Rapeseed meals from yellow- and black-seeded *B. napus* were produced using a high-temperature press, degreasing with petroleum ether. The chemical compositions of seed meals from yellow- and black-seeded rapeseeds are listed in Table 1. Seed meal derived from yellow-seeded *B. napus* presented higher protein (35.46% *vs.* 30.29% DM) and higher sucrose (7.85% *vs.* 7.29% DM). Significant differences in protein and sucrose levels were identified between yellow- and black-seeded *B. napus* seed meals (*p* < 0.05). Proteins are the main nutrients in rapeseed meal, supplying energy for metabolism after animal absorption. Improved protein content in yellow-seeded rapeseed meal indicates higher nutritional value for animal feeding. Theodoridou *et al.*, reported easier animal assimilation of yellow-seeded rapeseed meal from *B. napus* than that of black-seeded rapeseed meal [2]. Dairy cattle fed with meal from yellow-seeded *B. juncea* and *B. napus* exhibited higher absorption efficiency of proteins in the rumen and intestines than when they were fed with meal from black-seeded *B. napus*. Higher intestinal digestibility of total metabolizable protein and lower degraded protein balance were reported when yellow-seeded rapeseed meal was consumed compared with black-seeded rapeseed meal. Thus, the increase in protein content of the novel yellow-seeded *B. napus* derived from somatic hybrids of *B. napus–S. alba* indicates improved nutritional value for animal feeding, especially in the actual absorbed protein. High sucrose levels would improve the digestibility of rapeseed meal because sucrose is a carbohydrate that can be easily digested. Theander *et al.,* reported the sucrose content in seed meal of *Brassica* to be approximately 6.2%–8.0% [8]. In the current study, yellow-seeded rapeseed meal showed higher sucrose levels than black-seeded rapeseed; this result is consistent with previous reports [12,17].

**Table 1:** Chemical composition of black- and yellow-seeded *B. napus* seed meal.

Component	*B. napus*	*B. napus*
	Black (% DM)	Yellow (% DM)
Moisture	4.02 ± 0.20	3.54 ± 0.08
Ash	4.55 ± 0.0 ^a^	3.65 ± 0.0 ^b^
Crude protein	30.29 ± 0.37 ^b^	35.46 ± 0.27 ^a^
Sucrose	7.29 ± 0.09 ^b^	7.85 ± 0.03 ^a^
Starch	1.60 ± 0.04 ^b^	2.44 ± 0.08 ^a^
Dietary fiber	34.63 ± 0.3 ^a^	26.19 ± 0.31 ^b^
Crude fiber	8.86 ± 0.15 ^a^	4.56 ± 0.59 ^b^
Phytic acid	5.60 ± 0.06 ^a^	4.98 ± 0.06 ^b^
Total polyphenols	2.82 ± 0.14	2.67 ± 0.04
Total glucosinolates (μmol/g)	28.19 ± 0.35 ^a^	22.18 ± 0.52 ^b^

Data represent the mean ± standard error (*n* = 3). ^a^ and ^b^ indicate a significant difference at *p* < 0.05 level.

Antinutrients have significantly hindered the use of rapeseed meal for animal feeding. In this study, total dietary fiber (26.19% *vs.* 34.63% DM), crude fiber (4.56% *vs.* 8.86% DM), and glucosinolates (22.18 μmol/g DM *vs.* 28.19 μmol/g DM) were significantly higher in black-seeded rapeseed meal than in yellow-seeded rapeseed meal (Table 1). The observed reduction of glucosinolates in yellow-seeded rapeseed meal was similar to the findings of Slominski *et al.* [12]. Bell reported that various products from hydrolysis of glucosinolates under heat or low pH conditions would be harmful to animals, which were further influenced by anatomical and physiological differences in the gastrointestinal tracts of animals [11]. Glucosinolates in seed meal are degraded and conjugated by gut bacteria, inducing thyromegaly and damage in the alimentary tract of monogastric animals [18]. This negative effect remarkably hindered the application of rapeseed meal in animal breeding. Thus, selection of rapeseeds with lower glucosinolate content would be more beneficial to animal feeding. Recently, researchers have also been interested in the potential health advantages of glucosinolate in *B. rapa* and *B. oleracea* that are associated to the antioxidant and anti-carcinogenic properties [19,20]. The decrease of total dietary fiber in the novel yellow-seeded rapeseed meal used in this study agreed with the results of Slominski *et al.*, who reported that the average content of total dietary fiber is 33.0% in black-seeded rapeseed meal, 34.1% in canola meal, and 27.9% in yellow-seeded rapeseed meal. Generally, yellow-seeded rapeseed meal exhibits lower total dietary fiber content than black-seeded rapeseed meal, and the content of total dietary fiber is negatively correlated to the protein content in rapeseed meal [10]. Moreover, Daun *et al.*, proposed that the thinner seed coat of yellow rapeseed accompanies reduction of fiber content in comparison with black seeds, and Theander *et al.*, also confirmed the lower fiber content in yellow-seeded rapeseed meal than black-seeded rapeseed meal [8,9]. In the present study, fiber was reduced in yellow-seeded rapeseed meal, which might improve the digestibility and assimilation of meals. Slominski *et al.*, inferred that the reduction of fiber content in yellow-seeded rapeseed meal was attributable to the lower contribution of the hull fraction to the total seed mass, larger seed size, and lower fiber content of the hull fraction [12].

The phytic acid (4.98% *vs.* 5.60% DM) and total polyphenol (2.67% *vs.* 2.82% DM) contents were lower in yellow-seeded rapeseed meal than in black-seeded rapeseed meal (Table 1). Phytic acid is another antinutrient in rapeseed meal. Fontaine *et al.*, reported that phytic acid conjugates with Ca, Mg, and K form phytate or phytate-protein complexes in soybean, which hindered the availability of essential mineral elements and decreased the digestibility of proteins [21]. Nwokolo *et al.*, also proposed that certain phytic acid concentrations significantly reduced the utilization of mineral elements, resulting in Zn or Ca deficiency and hypofunction in animals [22]. Furthermore, phytic acid significantly affected amylase, pepsin, and trypsin activities during digestion of soybean meal [23]. Phytate can change Na partitioning and influence the Na-dependent transport of glucose and peptides in the gut [24]. The different amounts of phytic acid between yellow- and black-seeded rapeseed meals contribute to their nutritional differences. Slominski has summarized the effects of phytate on the growth and nutrient utilization of broiler chickens and laying hens [25].

Profiles of essential amino acids and total amino acids are listed in Table 2. Yellow-seeded rapeseed meal contained more essential amino acids than black-seeded *B. napus*, including serine (5.86 mg/g *vs.* 2.65 mg/g), methionine and cysteine (1.36 mg/g *vs.* 0.21 mg/g), isoleucine (0.33 mg/g *vs.* 0.20 mg/g), glycine (0.62 mg/g *vs.* 0.39 mg/g), alanine (2.95 mg/g *vs.* 1.06 mg/g), phenylalanine (2.08 mg/g *vs.* 1.26 mg/g), histidine (0.98 mg/g *vs.* 0.50 mg/g), lysine (1.65 mg/g *vs.* 0.80 mg/g), and arginine (1.59 mg/g *vs.* 1.17 mg/g) (*p* < 0.05) (Table 2). In terms of total amino acids, phenylalanine (8.17 mg/g *vs.* 7.06 mg/g), lysine (9.52 mg/g *vs.* 9.02 mg/g), threonine (9.37 mg/g *vs.* 8.29 mg/g), isoleucine (13.39 mg/g *vs.* 11.97 mg/g), asparagine (12.91 mg/g *vs.* 11.71 mg/g), glutamic acid (23.75 mg/g *vs.* 21.18 mg/g), methionine and cysteine (32.15 mg/g *vs.* 29.01 mg/g), tyrosine (15.44 mg/g *vs.* 13.80 mg/g), glycine (2.28 mg/g *vs.* 1.83 mg/g), alanine (6.99 mg/g *vs.* 6.04 mg/g), histidine (6.73 mg/g *vs.* 5.88 mg/g), and arginine (12.13 mg/g *vs.* 10.44 mg/g) were significantly higher in yellow-seeded rapeseed meal than in black-seeded rapeseed meal (Table 2). Methionine and cysteine are sulfur-containing amino acids considered as limiting amino acids. Rapeseed meal contains more sulfur amino acids than soybean meal, indicating comparative nutritional value of rapeseed and soybean meal [17]. Khajali and Slominski reported that canola meal, which contains more methionine and cysteine but less lysine, compares favorably with soybean meal in terms of amino acid content [4]. Moreover, the essential amino acid content (*i.e.*, phenylalanine, threonine, and isoleucine) directly affects the nutritional value of seed meal [26]. Improvement of both limiting and essential amino acids in yellow-seeded rapeseed meal suggests its potential application for animal feeding. Jia *et al.,* showed that the digestibility of total amino acids was higher in chickens fed with yellow-seeded rapeseed meal than in those fed with black-seeded rapeseed meal [27].

**Table 2:** Free and total amino acid composition of black- and yellow-seeded *B. napus* seed meal.

Amino Acid	Free Amino Acid		Total Amino Acid	
	*B. napus*	*B. napus*	*B. napus*	*B. napus*
	Yellow (mg/g)	Black (mg/g)	Yellow (mg/g)	Black (mg/g)
Asparagine	6.42 ± 0.11	6.67 ± 0.33	12.91 ± 0.28 ^a^	11.71 ± 0.08 ^b^
Threonine	0.44 ± 0.01	0.45 ± 0.01	9.37 ± 0.25 ^a^	8.29 ± 0.14 ^b^
Serine	5.86 ± 0.03 ^a^	2.65 ± 0.22 ^b^	8.27 ± 0.34	7.50 ± 0.22
Glutamic	15.5 ± 0.11	15.5 ± 0.55	23.75 ± 0.25 ^a^	21.18 ± 0.41 ^b^
Methionine + Cysteine	1.36 ± 0.18 ^a^	0.21 ± 0.01 ^b^	32.15 ± 0.47 ^a^	29.01 ± 0.33 ^b^
Isoleucine	0.33 ± 0.04 ^a^	0.20 ± 0.0 ^b^	13.39 ± 0.36 ^a^	11.97 ± 0.21 ^b^
Leucine	0.34 ± 0.04	0.28 ± 0.04	2.52 ± 0.04	2.23 ± 0.16
Tyrosine	1.22 ± 0.09	1.15 ± 0.04	15.44 ± 0.32 ^a^	13.80 ± 0.27 ^b^
Glycine	0.62 ± 0.02 ^a^	0.39 ± 0.02 ^b^	2.28 ± 0.10 ^a^	1.83 ± 0.04 ^b^
Alanine	2.95 ± 0.03 ^a^	1.06 ± 0.0 ^b^	6.99 ± 0.22 ^a^	6.04 ± 0.09 ^b^
Valine	0.70 ± 0.12	0.51 ± 0.03	4.25 ± 0.20	3.69 ± 0.02
phenylalanine	2.08 ± 0.01 ^a^	1.26 ± 0.05 ^b^	8.17 ± 0.23 ^a^	7.06 ± 0.00 ^b^
Histidine	0.98 ± 0.09 ^a^	0.50 ± 0.04 ^b^	6.73 ± 0.21 ^a^	5.88 ± 0.05 ^b^
Lysine	1.65 ± 0.01 ^a^	0.80 ± 0.01 ^b^	9.52 ± 0.19	9.02 ± 0.03
Arginine	1.59 ± 0.05 ^a^	1.17 ± 0.02 ^b^	12.13 ± 0.42 ^a^	10.44 ± 0.06 ^b^

Data represent the mean ± standard error (*n* = 3). ^a^ and ^b^ indicate a significant difference at *p* < 0.05 level.

In the present study, yellow-seeded rapeseed meal showed higher mineral element content than black-seeded rapeseed meal, including Fe (91.02 mg/kg *vs.* 87.48 mg/kg), Mn (48.12 mg/kg *vs.* 37.63 mg/kg), Zn (74.89 mg/kg *vs.* 54.82 mg/kg), Cu (4.96 mg/kg *vs.* 4.67 mg/kg), and K (12,161.50 mg/kg *vs.* 12,157.70 mg/kg), and the differences of Fe, Mn, and Zn contents between the two types of rapeseed meal were significant (*p* < 0.05). By contrast, the amounts of P (10,999.92 mg/kg *vs.* 11,966.29 mg/kg), Ca (3990.40 mg/kg *vs.* 5645.36 mg/kg), and Mg (3767.20 *vs.* 3860.59 mg/kg) were lower in yellow-seeded rapeseed meal than in black-seeded rapeseed meal (Table 3). Tenore *et al.* have previously reported similar content of minerals in *B. rapa* seed meal [28].

**Table 3:** The element composition of black- and yellow-seeded *B. napus* seed meal.

Component	*B. napus*	*B. napus*
	Black (mg/kg)	Yellow (mg/kg)
Cu	4.67 ± 0.16	4.96 ± 0.24
Fe	87.48 ± 0.4 ^b^	91.02 ± 0.19 ^a^
Mn	37.63 ± 0.59 ^b^	48.12 ± 0.37 ^a^
Zn	54.82 ± 0.41 ^b^	74.89 ± 0.42 ^a^
K	12,157.70 ± 72.3	12,161.50 ± 176.9
P	11,966.29 ± 56.9 ^a^	10,999.92 ± 108.9 ^b^
Ca	5645.36 ± 38.42 ^a^	3990.40 ± 64.24 ^b^
Mg	3860.59 ± 26.6 ^a^	3767.20 ± 70.3 ^b^

Data represent the mean ± standard error (*n* = 3). ^a^ and ^b^ indicate a significant difference at *p* < 0.05 level.

### 2.2. Comparison of Polyphenolics between Yellow- and Black-Seeded Rapeseed Meals

On the basis of the profiles of polyphenolics in rapeseed [29], we compared the phenolic contents in the seed meal of the two rapeseed lines. Up to 15 chemicals were identified through HPLC-DAD analysis (Table 4). Compared with black-seeded rapeseed meal, kaempferol-3-*O*-glucoside, kaempferol-3-*O*-diglucoside, kaempferol-3-*O*-sinapoylsophoroside, isorhamnetin-3-*O*-glucoside, isorhamnetin-*O*-glucoside-sulfate, isorhamnetin-3-*O*-glucoside-7-*O*-glucoside, quercetin-3-*O*-glucoside, and quercetin-3-*O*-diglucoside-7-*O*-glucoside were reduced in yellow-seeded rapeseed meal. However, sinapine, *cis*-sinapic acid, *trans*-sinapic acid, procyanidin B2, isorhamnetin-3-*O*-glucoside-7-*O*-acetylglucoside, km-3-*O*-sinapoyldiglucoside-7-*O*-sinapoylglucoside, and quercetin-3-*O*-sophoroside were higher in yellow-seeded rapeseed meal than in black-seeded rapeseed meal. Jakobek *et al.*, reported that phenolics prefer to conjugate with lipids and proteins, thus, the reduction of polyphenolics in yellow-seeded rapeseed meal improves the quality of rapeseed meal and might help improve the assimilation of energy in seed meal [30,31]. Cartea *et al.*, reviewed that flavonoids and non-flavonoids are commonly conjugated to sugars and organic acids [32]. Besides, phenolic compounds (condensed tannins) in rapeseed meal are able to form soluble and insoluble complexes with polysaccharides and other macromolecules and reducing their bioactivity [33]. Flavonoids in seed meal are poorly absorbed by animals due to the naturally occurring glycosides sugar moieties elevate the hydrophilicity, and no enzyme have found to split the glycoside bond [34]. Thus, the reduction of phenolics in meal of yellow rapeseed would be more valuable to animal feeding.

## 3. Experimental Section

### 3.1. Plant Materials

Seeds of yellow-seeded *B. napus* (line W82) derived from the progeny of *B. napus–S. alba* hybrids and black-seeded rapeseed (*B. napus* cv. “Yangyou 6”) were used in this study [35,36]. “Yangyou 6” was the parental line used for successive back-crossing of somatic hybrids. After removing oil with the conventional prepress solvent extraction process described by Slominski *et al.* [37], seed meal was then extracted twice using 25 mL of petroleum ether (30 min for each extraction) at 30–60 °C, aiming to remove the residual oil in seed meal. After drying under the fumehood, the samples were ground into powder to pass through a 1 mm sieve, and finally, the meals were subjected to component analysis.

**Table 4:** LC/MS data of tentatively identified polyphenolics in meal of black- and yellow-seeded *B. napus* and their quantitative analysis using DAD at 280 and 330 nm.

Peak	Compound	Content (μg/g)		Mass Spectrum (*m*/*z*)		MS^2^ [M − H]^−^ (*m*/*z*)	MS^2^ [M + H]^+^ (*m*/*z*)	UV λ_max_ (nm)
		Black	Yellow	[M − H]^−^	[M + H]^+^			
1	sinapine	5469.73 ± 145.78 ^b^	8309.9 ± 125.54 ^a^	-	310	-	119, 175, 207, 251	nd
2	cis-sinapic acid	371.5 ± 35.2 ^b^	465.49 ± 43.32 ^a^	223	-	193, 164	-	nd
3	trans-sinapic acid	4439.24 ± 89.08 ^b^	8706.89 ± 154.39 ^a^	223	-	208, 179, 164	-	nd
4	km-3-*O*-glucoside	15.36 ± 0.34 ^a^	7.98 ± 0.04 ^b^	447	-	357, 285, 284	-	265, 345
5	km-3-*O*-diglucoside	14.53 ± 0.65 ^a^	6.2 ± 0.02 ^b^	609	-	447, 285	-	265, 335
6	km-3-*O*-sinapoylsophoroside	27.34 ± 0.78 ^a^	16.06 ± 0.97 ^b^	815	-	623, 609, 591	-	nd
7	km-3-*O*-sinapoyldiglucoside-7-*O*-sinapoylglucoside	57.71 ± 2.56 ^a^	63.96 ± 2.63 ^a^	1183	-	815, 609	-	270, 330
8	is-3-*O*-glucoside	34.18 ± 2.43 ^a^	17.44 ± 1.07 ^b^	477	-	315, 314	-	265, 355
9	is-*O*-glucoside-sulfate	4.29 ± 0.04 ^a^	2.67 ± 0.02 ^b^	557	-	477, 395, 315	-	265, 320
10	is-3-*O*-glucoside-7-*O*-glucoside	17.74 ± 0.56 ^a^	1.77 ± 0.00 ^b^	639	-	477, 315	-	nd
11	is-3-*O*-glucoside-7-*O*-acetylglucoside	16.58 ± 0.54 ^b^	21.89 ± 0.83 ^a^	681	-	476, 315	-	nd
12	qn-3-*O*-glucoside	596.84 ± 34.78 ^a^	492.51 ± 37.62 ^b^	463	-	300	-	nd
13	qn-3-*O*-sophoroside	21.11 ± 0.35 ^a^	22.8 ± 3.24 ^a^	625	-	463, 445, 300	-	265, 345
14	qn-3-*O*-diglucoside-7-*O*-glucoside	22.16 ± 2.02 ^a^	6.14 ± 0.38 ^b^	787	-	625	-	nd
15	procyanidin B2 (DP [2])	15.56 ± 0.89 ^b^	68.1 ± 3.87 ^a^	577	-	425, 407, 289	-	nd

Abbreviations: km, kaempferol; is, isorhamnetin; qn, quercetin; nd, not detected. Data represent the mean ± standard error (*n* = 3). ^a^ and ^b^ indicate a significant difference at *p* < 0.05 level.

### 3.2. Chemical Analysis of Seed Meal

For determination of protein contents, a nitrogen analyzer was used to obtain the content of N, and the content of crude protein was calculated by N × 6.25. Seed meal (0.5 g) was treated with 6 N HCl (20 mL) at 110 °C for 24 h, and total amino acid content was then determined using ninhydrin colorimetry and an Biochrom 30 amino acid analyzer (Biochrom Ltd., Cambridge, UK). For free amino acid analysis, the seed meal (0.5 g) was extracted with picric acid (20 mL) then determined on the amino acid analyzer [38]. Total dietary fiber was determined in accordance with the standard procedures of the Association of Official Analytical Chemists (AOAC, 1998); crude fiber was determined following the AOAC (2000) procedures 962.09. The AOAC (2005) procedures were used for dry matter (930.15) and ash (942.05) determination. Starch was analyzed using the standard AOAC (2000) procedure 996.11 [39]. Phytic acid was determined referring to Thompson *et al.* [40]. Seed meal (0.5 g) was extracted with 100 g/L Na_2_SO_4_ (5 mL) in 30 g/L trichloroacetic acid (5 mL). After shaking for 2 h at room temperature, the suspension was collected after centrifugation at 13,000 g for 10 min. The pellet was re-extracted and all the suspension was combined. The phytic acid was precipitated with 0.3 g/L FeCl_3_ (2 mL), and its amount was calculated according to the difference of phosphorus value between the initial supernatant and the supernatant after precipitated with FeCl_3_. A conversion factor of 3.55 from phosphorus and phytic acid was used. Glucosinolates were determined via near-infrared spectroscopy, which developed a new calibration to measure major fraction, and no external standards were needed [5]. The content of sucrose was determined by traditional anthrone colorimetry. Minerals were determined using the method previously described by Tenore *et al.* [28]. Inductively coupled plasma quadrupole mass spectrometry (ICP-QMS) (ElanDRC II, Perkin-Elmer SCIEX, Norwalk, CT, USA) was used. The operational parameters were set according to Tenore *et al.* [28]. Calibrations of Cu, Fe, Mn, Zn, K, P, Ca and Mg were carried out for quantification of mineral elements in meal of yellow and black seed.

### 3.3. Analysis of Phenolics in Seed Meal

Phenolics were extracted using the method previously described by Shao *et al.* with some modifications, and the extracts were filtered through 0.45 μm Teflon membranes (Interchim, Montlucon, France) and then used for high-performance liquid chromatography (HPLC)-electrospray ionization (ESI)/tandem mass spectrometry (MS^2^) analysis [32]. Seed meal (0.5 g) was extracted with 80% methanol (3 mL) in an ultrasonic bath at 4 °C for 1 h. The supernatant was collected after centrifugation at 10,000 rpm for 10 min at 4 °C. After re-extraction of the pellet, all supernatants were combined and then concentrated under vacuum. The crude extracts were diluted with methanol (0.5 mL), and filtered through a 0.45 μm Teflon membrane for further analysis. Total phenol content was determined using the method previously described by Jiang *et al.* [7]: extract (50 μL) was added to distilled water (50 μL) and Folin-Ciocalteu reagent (100 μL), and then incubated for 3 min at room temperature before Na_2_CO_3_ (50 μL) was added. The total phenol content was reported as (−)-epicatechin equivalents based on a calibration curve. Using (−)-epicatechin as standard, the absorbance was measured at 725 nm after the incubation for 45 min to acknowledge the content of phenol content. The total phenol content was reported as (−)-epicatechin equivalents based on a calibration curve. HPLC analysis of 10 μL extracts was performed on an Agilent 6460 instrument (Agilent Technologies Co. Ltd., Palo Alto, CA, USA) equipped with automatic injector, quaternary pump, thermostatted column compartment, and diode array detector (DAD). The Agilent Masshunter Qualitative Analysis software was used for data collection and analysis. LC/MS data of tentatively identified polyphenolics in meal of black- and yellow-seeded *B. napus* and their quantitative analysis using DAD at 280 and 330 nm. Samples were separated using XB-C18 column (2.1 mm × 150 mm, 3.5 μm internal diameter, Ultimate, Welch, USA), with a flow rate of 0.3 mL/min, oven temperature of 30 °C, and a detection range of 190–800 nm. The mobile phase consisted of a combination of solvent A (0.1% formic acid in water, *v*/*v*) and solvent B (0.1% formic acid in acetonitrile, *v*/*v*). The linear gradient was as follows: 10% B (*v*/*v*) for 5 min, 10%–90% B for 40 min, 90% B for 10 min, 90%–10% B for 1 min, and hold at 10% B for 10 min.

Tandem mass spectrometry was operated in positive and negative ESI modes. The mass spectrometer used in phenolic analysis was MS QQQ coupled in LC/MC system (Agilent Technologies, Waldbronn, Germany). The parameters were as follows: drying gas temperature = 300 °C, drying gas flow = 10 L/min, nebulizer gas (N_2_) pressure = 15 psi, sheath gas temperature 250 °C, sheath gas flow = 7 L/min, capillary voltage = 4000 V, and spraying voltage = 500 V. Full scan spectra of 500 ms and an *m/z* range of 90–2000 were selected. MS^2^ fragmentation patterns of phenolics were determined under 15–40 V, using the Agilent MassHunter Workstation Data Acquisition and Agilent MassHunter Qualitative Analysis software. Procyanidin B2, (−)-epicatechin, sinapine, and quercetin-3-*O*-β-d-glucoside, which were purchased from Sigma (St. Louis, MO, USA), and isorhamnetin-3-*O*-glucoside, which was purchased from ChromaDex (Irvine, CA, USA), were used as standards for phenolic quantification. Since not all the standards of the chemicals identified in the Table 4 were commercially available, we alternatively used the standards for a group of chemicals with similar parent compound. Phenolics could be classified into four types, including procyanidin B2, (−)-epicatechin, sinapine, flavonols (including quercetin, isorhamnetin and kaempferol). Procyanidin B2, (−)-epicatechin, sinapine, and quercetin-3-*O*-β-d-glucoside were adequate for quantification of all the chemicals, of which quercetin-3-*O*-β-d-glucoside was used as standard for quercetin, isorhamnetin and kaempferol. Statistical analyses of the contents were expressed as mean ± standard error of samples from three independent extractions. A significance level of *p* < 0.05 was considered.

## 4. Conclusions

Rapeseed meal is abundant in protein compared with other seed meals, making it optimal for animal feeding. However, antinutrients in rapeseed meal limit its application. Breeding of yellow-seeded *B. napus* improves the quality of rapeseed meal. In this study, a novel yellow-seeded line of *B. napus* derived from a somatic *B. napus–S. alba* hybrid was shown to be of greater nutritional value compared with black-seeded rapeseed, that is, yellow-seeded rapeseed meal exhibited more nutrient substances and less antinutrients than black-seeded rapeseed meal. The chemical composition of rapeseed meal, in which proteins are the resources for animal feeding, but the antinutrients influence the actual digestibility and assimilation of the nutrients in rapeseed meal is rather complicated. Thus, further animal feeding experiments using seed meals from both yellow- and black-seeded rapeseeds are necessary to evaluate the actual value of yellow-seeded rapeseed meal.
